# Prognostic Role of High-Grade Tumor Budding in Pancreatic Ductal Adenocarcinoma: A Systematic Review and Meta-Analysis with a Focus on Epithelial to Mesenchymal Transition

**DOI:** 10.3390/cancers11010113

**Published:** 2019-01-19

**Authors:** Rita T. Lawlor, Nicola Veronese, Alessia Nottegar, Giuseppe Malleo, Lee Smith, Jacopo Demurtas, Liang Cheng, Laura D. Wood, Nicola Silvestris, Roberto Salvia, Aldo Scarpa, Claudio Luchini

**Affiliations:** 1 ARC-Net Research Center, University and Hospital Trust of Verona, 37134 Verona, Italy; ritateresa.lawlor@univr.it; 2 National Institute of Gastroenterology-Research Hospital, IRCCS “S. de Bellis”, 70013 Castellana Grotte, Italy; ilmannato@gmail.com; 3 Department of Surgery, Section of Pathology, San Bortolo Hospital, 36100 Vicenza, Italy; alessia.nottegar@gmail.com; 4 Department of General and Pancreatic Surgery, The Pancreas Institute, University and Hospital Trust of Verona, 37142 Verona, Italy; giuseppe.malleo@univr.it (G.M.); roberto.salvia@univr.it (R.S.); 5 Cambridge Centre for Sport and Excercise Sciences, Anglia Ruskin University, Cambridge CB1 1PT, UK; Lee.Smith@anglia.ac.uk; 6 Primary Care Department, Azienda USL Toscana Sud Est, 58100 Grosseto, Italy; eritrox7@gmail.com; 7 Department of Pathology and Laboratory Medicine, Indiana University School of Medicine, Indianapolis, IN 46202, USA; liang_cheng@yahoo.com; 8 Department of Pathology, The Sol Goldman Pancreatic Cancer Research Center, The Johns Hopkins University School of Medicine, Baltimore, MD 21231, USA; ldelong1@jhmi.edu; 9 Medical Oncology Unit, IRCCS Cancer Institute “Giovanni Paolo II” of Bari, 70124 Bari, Italy; silvestrisnicola@gmail.com; 10 Department of Diagnostics and Public Health, Section of Pathology, University of Verona, 37134 Verona, Italy; claudio.luchini@univr.it

**Keywords:** budding, buds, epithelial to mesenchymal transition, EMT, pancreatic cancer

## Abstract

This study aims at clarifying the prognostic role of high-grade tumor budding (TB) in pancreatic ductal adenocarcinoma (PDAC) with the first systematic review and meta-analysis on this topic. Furthermore, we analyzed with a systematic review the relationship between TB and a recently suggested TB-associated mechanism: the epithelial to mesenchymal transition (EMT). Analyzing a total of 613 patients, 251 of them (40.9%) with high grade-TB, we found an increased risk of all-cause mortality (RR, 1.46; 95% CI, 1.13–1.88, *p* = 0.004; HR, 2.65; 95% CI, 1.79–3.91; *p* < 0.0001) and of recurrence (RR, 1.61; 95% CI, 1.05–2.47, *p* = 0.03) for PDAC patients with high-grade TB. Moreover, we found that EMT is a central process in determining the presence of TB in PDAC. Thanks to this meta-analysis, we demonstrate the potential clinical significance of high-grade TB for prognostic stratification of PDAC. TB also shows a clear association with the process of EMT. Based on the results of the present study, TB should be conveyed in pathology reports and taken into account by future oncologic staging systems.

## 1. Introduction

Pancreatic ductal adenocarcinoma (PDAC) is an aggressive tumor that represents the seventh cause of death for cancer in the world, with an increasing tendency of incidence for the future [1,2,3,4]. 

Notably, the majority of patients present with locally advanced or metastatic disease, and are thus not eligible for surgical resection with curative intent. For patients with resectable cancers, robust morphological and molecular predictors able to stratify PDAC prognosis are still needed. In fact, following the classical tumor, nodes, metastasis (TNM) parameters [5,6], few histological features have been demonstrated of reliable prognostic significance: resection margin status, lymphovascular invasion, perineural invasion and extranodal extension of nodal metastasis [6,7,8,9].

**Figure 1:**
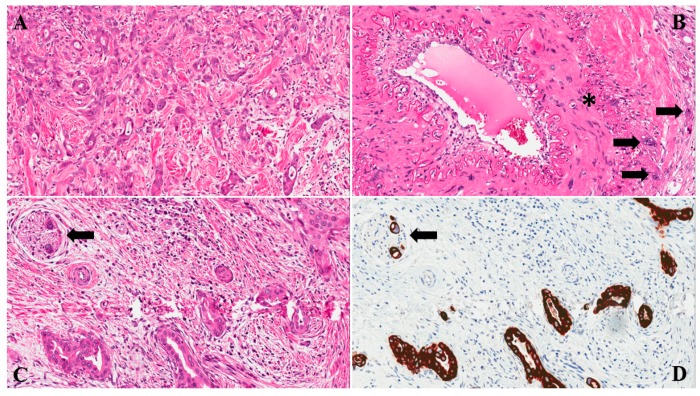
A classical example of PDAC with high-grade tumor budding is shown: note the high number of neoplastic cell clusters formed by less than 5 cells (original magnification: 20×) (**A**); PDAC with high-grade tumor budding can invade the surrounding tissue also with a single cell pattern: few single cancer cells (black arrows) invading the wall of an arteriolar vessel (asterisk, original magnification: 20×) (**B**), and two single PDAC cells of (black arrow) invading a nerve (original magnification: 20×), (**C**): hematoxylin-eosin, (**D**): immunohistochemical staining of the same field of Figure 1C with cytokeratin 8/18 staining.

One morphological feature recently suggested as a possible reliable marker of poor prognosis in PDAC is tumor budding (TB). It is defined as the presence of a single cell or cell clusters composed of up to 5 neoplastic cells at the infiltrative front of the tumor, and is an indicator of cancer invasiveness (Figure 1) [10,11]. This histological parameter has been well studied in colorectal cancer, but its independent prognostic significance has also been demonstrated for other cancer types [10,11,12,13,14,15,16]. 

Since the clinical significance of TB has not yet been fully clarified in PDAC, we aim to study its prognostic role with the first systematic review and meta-analysis on this topic. Furthermore, since some authors have suggested that TB could represent one of the morphological hallmarks of epithelial-to-mesenchymal transition (EMT) [17,18,19], we aim at providing some insights on this process and its association with TB in PDAC, using a systematic review-based approach.

## 2. Results

### 2.1. Search Results

Altogether, 131 non-duplicated articles were identified through the literature search. After excluding 100 articles based on title/abstract review, 31 articles were retrieved for full text review and, following the application of the inclusion criteria, six unique articles were considered as eligible for the meta-analysis (Figure S1) [20,21,22,23,24,25].

### 2.2. Study and Patient Characteristics

Almost all cases of PDAC in the selected manuscripts showed the presence of tumor budding, thus the authors distinguished patients with high grade-TB vs. low grade-TB. The grade of TB was established based on the number of tumor buds (e.g., high grade-TB in case of at least 10 buds per high power field, Table S1). Due to the modality of TB assessment in such manuscripts and since meta-analyses have to reflect the primary studies, we analyzed the prognostic significance of high grade-TB vs. low grade-TB in PDAC. Altogether, the studies followed-up 613 patients, 251 (40.9%) of which had high grade-TB. All the selected studies were published in the last 5 years. They involved distinct cohorts of patients covering almost all continents (Asia, America, Europe and Africa). The median Newcastle-Ottawa Scale (NOS) score was 7 points (range: 6–8), with no manuscript considered as at high risk of bias (Tables S1 and S2). Two studies reported raw data only (e.g., number of deaths) [22,24] whilst in the 4 studies reporting adjusted analyses, the median number of confounders considered in the multivariate analysis was 4 (Tables S1 and S3) [20,21,23,25]. There was no statistically significant difference between high grade-TB and low grade-TB groups of patients regarding presence of lymph node metastasis or pathologic T stage.

### 2.3. Association between High Grade vs. Low Grade Tumor Budding and Survival

The outcomes analyzed in this meta-analysis were those reported in at least three studies: they were all-cause mortality (both RR and HR were provided) and risk of recurrence (HR only). 

#### 2.3.1. All-Cause Mortality (ACM)

**Figure 2:**
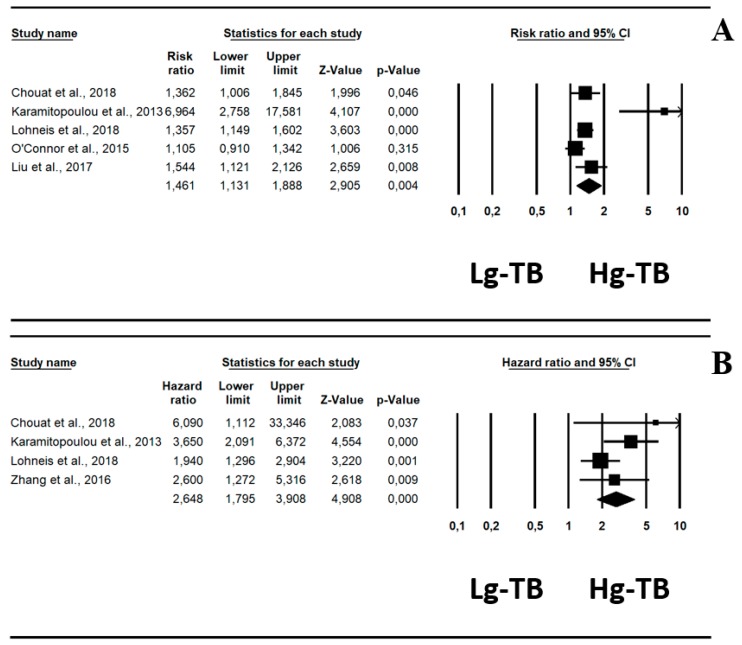
Forrest plot indicating pancreatic ductal adenocarcinoma risk ratio (RR, **A**) in pancreatic ductal adenocarcinoma and hazard ratio (HR, **B**) for all-cause mortality of patients with high-grade tumor budding (Hg-TB) vs. low-grade tumor budding (Lg-TB).

Pooling data from five studies [20,21,22,23,24], there was an increased RR of ACM in patients with high grade-TB, which was statistically significant (RR, 1.46; 95% CI, 1.13–1.88, *p* = 0.004, I^2^ = 76%) (Table 1, Figure 2A). In four studies reporting adjusted data from multivariate analysis [20,21,23,25], high grade-TB carried a significantly higher HR of ACM compared to low grade-TB, increasing its statistical significance (HR, 2.65; 95% CI, 1.79–3.91; *p* < 0.0001; I^2^ = 31%) (Table 1, Figure 2B).

#### 2.3.2. Risk of Recurrence (ROR)

Pooling data from three studies [21,22,23], the presence of high grade-TB increased the RR of ROR with statistical significance (RR, 1.61; 95% CI, 1.05–2.47, *p* = 0.03, I^2^ = 85%) (Table 1, Figure S2). 

### 2.4. Publication Bias and Meta-Regression Analyses

There was no risk of publication bias for all the analyzed indexes: RR of ACM (Egger’s test, 3.61 ± 1.41, *p* = 0.08), HR for ACM (Egger’s test, 1.85 ± 1.30; *p* = 0.29) and RR for ROR (Egger’s test, 4.13 ± 0.59, *p* = 0.09). There was high heterogeneity for RR of ACM and of ROR. However, since high heterogeneity was no longer reported once one outlier study was removed [20], appearing to be the main moderator of such heterogeneity, a meta-regression analyses were not performed.

### 2.5. Focus on Epithelial to Mesenchymal Transition

Using a systematic review-based approach, we found six different manuscripts that simultaneously analyzed TB and EMT in pancreatic ductal adenocarcinoma [20,22,26,27,28,29]. Their main findings have been summarized in Table 2. In all these manuscripts, we found specific expression patterns of EMT-associated markers, indicating that EMT is a central process in determining the presence of TB. Indeed, some studies showed an increased expression of mesenchymal markers such as vimentin [20,29], ZEB1 and ZEB2 [27] in TB cells, whereas other studies indicated a reduced or focal expression of epithelial markers, like E-cadherin [27,28,29], cytokeratin [22] and p63 [26].

## 3. Discussion

In this manuscript regarding the prognostic role of TB in PDAC, we found a statistically significant association between the presence of a high grade-TB and an increased risk of mortality and/or recurrence of disease. These findings appear reliable, also considering that the association of high grade-TB and risk of mortality has been confirmed using multivariable analyses data. The independent prognostic value of high grade-TB was confirmed also observing that both patients with or without high grade-TB showed no significant differences either in terms of presence of lymph node metastasis or of pathologic T stage. 

The low inclusion rate (4.6%) of papers after screening of the literature underlines the rigorous method applied for selection of manuscripts. In a meta-analysis, the inclusion rate of manuscripts after screening with the selected search strategy can vary greatly for a number of reasons. A low inclusion rate can occur when using a wide and all- encompassing search strategy to find all the papers on a specific topic, coupled with very strict criteria for paper selection. In our meta-analysis, very specific and strict criteria were applied, including: (i) presence of a clear comparison of prognostic factors between high grade-tumor budding (TB) vs. low grade-TB, and (ii) presence of histological diagnosis of PDAC with clear microscopic demonstration of TB among other criteria. Thus, the low inclusion rate indicates the high-quality search strategy for paper adherence and selection.

To further corroborate our results, no risk of publication bias emerged from all the analyzed indexes. In the second part of this study, we performed a systematic review to clarify the biological role of EMT in PDAC, and found that EMT plays a central role in PDAC with high grade-TB.

Since TB has shown a significant prognostic value in PDAC, it is also important to analyze the modalities to detect this morphological parameter during routine histological analyses. O’Connor and colleagues have compared the use of standard hematoxylin/eosin (H&E) staining vs. cytokeratin, reporting that the assessment of TB with H&E is reliable and can be only slightly improved by coupling it with immunohistochemical analysis. This specific insight demonstrates that TB can be easily assessed on routine histological sections and without additional exams, highlighting the feasibility of documenting TB on standard pathology reports. Notably, the recently proposed ITBCC method for TB scoring suggests the use of H&E sections. This is a standardized and well-designed scoring system, based on a Consensus Conference and validated for TB scoring in PDAC [30]. This scoring system is easily reproducible, designates assessing TB in the densest budding area at ×20 magnification (one hot-spot, 0.785 mm^2^), and subgroups cases into four categories: BD0: no buds; BD1: one to four buds; BD2: five to nine buds; and BD3: ≥10 buds. Its application can facilitate TB reporting in diagnostics and in the microscopic description of routine histological exams.

We also found that TB is a morphological aspect intimately associated with EMT, which is a process of biological changes that permits to epithelial cells to acquire the so-called “mesenchymal phenotype” [31]. This phenotype leads to a reorganization of cytoskeletal and cell-surface proteins, leading to increased invasiveness and migratory capacities and to an acquisition of elevated resistance to apoptosis; it is also characterized by loss of cell polarity, down-regulation of E-cadherin and acquisition of mesenchymal markers and related transcription factors (e.g., Vimentin, Twist1, Snail2, ZEB1, ZEB2) [31,32,33]. All six manuscripts we identified through a systematic review on this specific topic showed statistically significant data indicating a clear association between the activation of EMT and the presence of high grade-TB in PDAC (Table 2). Indeed, while PDAC with high grade-TB demonstrated an increased expression of mesenchymal markers such as vimentin [20,29], ZEB1 and ZEB2 [27], PDAC with low grade-TB showed a reduced or focal expression of epithelial markers, like E-cadherin [27,28,29], cytokeratin [22] and p63 [26]. In particular, the EMT markers most used in this context are Cytokeratin AE1/AE3 and E-Cadherin as epithelial markers and Vimentin as mesenchymal markers. EMT and TB are also strongly associated in other tumor types [34,35,36,37]: this indicates that TB is the morphological manifestation of EMT, and that EMT is an important biological mechanism shared by those tumors that invade the surrounding tissue through single cells or small cell clusters.

We demonstrated that high grade-TB can help in stratifying PDAC prognosis. Of interest, Wartenberg and colleagues generated new insights on this topic in a recent manuscript, coupling the analysis of histological parameters (including TB) with their molecular profiling and with the analysis of the types of tumor-associated inflammatory cells [26]. Three PDAC subtypes were identified, where the one with the highest grade of TB showed the worst outcome. This PDAC subtype, called “immune-escape” by the authors, presented reduced infiltration of T and B cells, was enriched in FOXP3 regulatory T cells, and harboured frequent *CDKN2A* and *SMAD4* mutations. Although further studies are needed to confirm the reliability of this PDAC categorization, the integration of the most important morphological parameters with immunological and molecular data may represent a key element to reach a better stratification of PDAC prognosis. Based on the results of the present study, TB should be taken into account in all these different but converging aspects.

## 4. Materials and Methods

This systematic review adhered to the Meta-analyses Of Observational Studies in Epidemiology (MOOSE) guidelines and Preferred Reporting Items for Systematic Reviews and Meta-Analyses (PRISMA) statement, following a predetermined protocol [38,39].

### 4.1. Inclusion and Exclusion Criteria

Studies were eligible for inclusion upon meeting the following criteria: (1) a prospective cohort or retrospective study design; (2) contained a comparison of prognostic factors between high grade-TB vs. low grade-TB; (3) contained histological diagnosis of PDAC with clear microscopic demonstration of TB; (4) contained data about mortality/recurrence of disease; (5) were published in a peer review journal or published abstract. We considered articles in any language. Exclusion criteria were: (1) no presence of PDAC, (2) no clear microscopic demonstration of TB, (3) no data about prognostic parameters in the title/abstract, (4) no comparison between high grade-TB vs. low grade-Tb patients, and (5) in vitro or animal studies.

### 4.2. Data Sources and Literature Search Strategy

Two investigators (R.T.L., C.L.) independently searched PubMed, Embase and SCOPUS until 30 September 2018. The search terms used in PubMed included combinations of the following keywords: (“pancreas” OR “pancreatic” OR “PDAC”) AND (“budding” OR “sprouting” OR “bud” OR “buds”) AND (“mortality” OR “mortalities” OR “fatality” OR “fatalities” OR “death*” OR “survival” OR “recurrence” OR “hazard ratio” OR “HR” OR “relative risk” OR “RR” OR “prognosis” OR “progression”). A similar research was made in SCOPUS. We considered the reference lists of all included articles and of previous related reviews.

### 4.3. Study Selection

Following the searches as outlined above, after removal of duplicates, two independent reviewers (A.N., N.V.) screened titles and abstracts of all potentially eligible articles. The two authors applied the eligibility criteria, considered the full texts, and a final list of included articles was reached through consensus with a third author (C.L.).

### 4.4. Data Extraction

Two authors were involved in data extraction in a standardized Microsoft Excel database. Specifically, one author (R.T.L.) extracted data from the included articles and a second independent author (C.L.) validated the data. For each article, we extracted information about authors, year of publication, country, exclusion criteria, methods of TB assessment, number of participants and gender, age, pathologic tumor stage, presence of lymph node metastasis, number of adjustments in survival analyses and duration of follow-up.

### 4.5. Outcomes

The primary outcomes were number of deaths and number of recurrences after treatment during the follow-up period in patients with high grade-TB vs. low grade-TB. Secondary outcomes were hazard ratios, adjusted for the maximum number of confounders available, regarding the same outcomes, taking those with low grade-TB as reference. A meta-analysis was conducted only in case of at least 3 studies per outcome.

### 4.6. Assessment of Study Quality 

We used the Newcastle-Ottawa Scale (NOS) to evaluate study quality [40]. The NOS provides an assessment of the methodological quality of observational studies and its content validity and reliability have been widely assessed [41]. Included studies are assessed on 8 items across three key areas: selection of the participants, comparability of the participants and outcomes. Two authors (C.L, N.V.) completed the NOS and each study receives an overall score for methodological quality of up to 9 points with a score of ≤5 (out of 9) indicating high risk of bias. 

### 4.7. Data Synthesis and Statistical Analysis 

All analyses were performed using Comprehensive Meta-Analysis (CMA) 2. In our primary analyses, pooled risk ratios (RRs) and 95% confidence intervals (CIs) of risk of mortality and of recurrence between high grade-TB vs. low grade-TB were calculated using DerSimonian-Laird random-effects models [42]. In secondary analyses, pooled hazard ratios (HRs) with 95% CIs adjusted for the maximum number of covariates available in the articles, were also calculated for providing additional information if the relationship between TB status and outcomes was influenced by potential confounders. Heterogeneity across studies was assessed by the I^2^ metric and chi square statistics [43]. In presence of significant heterogeneity (*p* < 0.05) after removing outlier studies, we planned to conduct a series of meta-regression analyses according to TB status and each of prognostic parameters considered. Finally, we investigated publication bias for our primary meta-analysis with a visual inspection of funnel plots coupled with Egger bias test [44].

### 4.8. Focus on Epithelial-to-Mesenchymal Transition with a Systematic Review-Based Approach

We modified the search strategy originally used for this meta-analysis to find all manuscripts investigating simultaneously TB and EMT-associated markers/parameters in PDAC. The search terms used in PubMed included combinations of the following keywords: (“pancreas” OR “pancreatic” OR “PDAC”) AND (“budding” OR “sprouting” OR “bud” OR “buds”) AND (“EMT” or “epithelial to mesenchymal”). A similar research was made in Embase and in SCOPUS. We considered the reference lists of all included articles and of previous related reviews.

## 5. Conclusions

High-grade TB has a potentially high clinical significance for the prognostic stratification of PDAC. Furthermore, TB shows a clear association with the process of EMT. The integration of the most important morphological parameters with immunological and molecular data may represent a key element to reach a better stratification of PDAC prognosis and to design more effective therapeutic strategies. The results of our study indicate that TB should be conveyed in pathology reports and taken into account by future oncologic staging systems.

## Figures and Tables

**Table 1:** Risk Ratio and Hazard Ratio for survival indexes of PDAC patients based on high grade-TB vs. low grade TB status.

Parameter	N Studies	Risk Ratio (95% CI)	*p*-Value	Heterogeneity (I^2^%); Tau^2^	Egger Test (*p*-Value)
ACM	5	1.46 (1.13–1.88)	0.004	76%; 0.06	3.61 (0.08)
**Parameter**	**N Cohorts**	**Hazard Ratio** **(95% CI)**	***p*-Value**	**Heterogeneity** **(I^2^%); Tau^2^**	**Egger Test** **(*p*-Value)**
ACM	4	2.65 (1.79–3.91)	<0.0001	31%; 0.05	1.85 (0.29)
**Parameter**	**N Cohorts**	**Risk Ratio** **(95% CI)**	***p*-Value**	**Heterogeneity** **(I^2^%); Tau^2^**	**Egger Test** **(*p*-Value)**
ROR	3	1.61 (1.05–2.47)	0.007	85%; 0.11	4.13 (0.09)

Abbreviations: PDAC: pancreatic ductal adenocarcinoma; TB: tumor budding; ACM: all-cause mortality; ROR: risk of recurrence; CI: confidence intervals.

**Table 2:** Studies that analysed EMT and TB in pancreatic ductal adenocarcinoma, with their main findings.

References	EMT and Other Important Variables	Main Findings
Chouat, 2018 [20]	Cytokeratin (AE1/AE3) and vimentin (IHC)	High grade-TB was significantly associated with an increased vimentin expression (*p* = 0.002).
Galván, 2015 [27]	E-cadherin, β-catenin, SNAIL1, ZEB1, ZEB2, N-cadherin, TWIST1 (IHC and mRNA-ISH)	TB cells showed increased levels of ZEB1 (*p* < 0.0001) and ZEB2 (*p* = 0.0119) and reduced E-cadherin (*p* < 0.0001) and β-catenin (*p* < 0.0001) expression compared with the main tumor.
Kohler, 2015 [28]	Cytokeratin (AE1/AE3), E-cadherin, β-catenin, vimentin (IHC)	TB cells showed reduced E-cadherin expression compared with the main tumor. E-cadherin and β-catenin showed reduced expression in the tumor periphery than in the tumor center (*p* < 0.050).
Lapshyn, 2016 [29]	E-cadherin, β-catenin, vimentin (IHC), morphology of cancer-associated fibroblasts	Mesenterico-portal venous tumor infiltration was significantly associated with loss of E-cadherin in tumor buds (*p* = 0.020), increased vimentin expression (*p* = 0.03) and activated cancer-associated fibroblasts morphology.
Liu, 2017 [22]	Cytokeratin (AE1/AE3) (IHC)	Expression of cytokeratin in tumor budding was significantly lower than in primary tumor (*p* = 0.001).
Wartenberg, 2018 [26]	p63 and “immune-cell” markers	Three PDAC subtypes were identified: the one with the highest grade of TB showed focal p63 expression, frequent *CDKN2A* and *SMAD4* mutations, a reduced presence of T and B cells, was enriched in FOXP3 regulatory T cells, and has the worst outcome.

Abbreviations: IHC: immunohistochemistry; ISH: in situ hybridization.

## Supplementary Materials

The following are available online at http://www.mdpi.com/2072-6694/11/1/113/s1, Table S1. Characteristics of the studies according to tumor budding, high-grade (Hg-TB) vs. low grade (Lg-TB), Table S2. Methodological quality of cohort studies included in the meta-analysis, Table S3. Type and number of adjustments (in addition of tumor budding) for each study, Figure S1. PRISMA checklist for this meta-analysis, Figure S2. Forrest plot indicating pancreatic ductal adenocarcinoma risk ratio for recurrence in patients with high-grade tumor budding vs. low-grade tumor budding.
