# Supplementary material for: Prognostic Role of High-Grade Tumor Budding in Pancreatic Ductal Adenocarcinoma: A Systematic Review and Meta-Analysis with a Focus on Epithelial to Mesenchymal Transition
Source: Cancers (Basel). 2019 Jan 19;11(1):113. doi: 10.3390/cancers11010113 (PMC6356259; doi:10.3390/cancers11010113)
Supplement: Supplementary file 1 [file cancers-11-00113-s001.pdf]

cancers

Supplementary Materials: Prognostic Role of High-Grade Tumor Budding in Pancreatic Ductal Adenocarcinoma: A Systematic Review and Meta-Analysis with a Focus on Epithelial to Mesenchymal Transition

Rita T. Lawlor, Nicola Veronese, Alessia Nottegar, Giuseppe Malleo, Lee Smith, Jacopo Demurtas, Liang Cheng, Laura D. Wood, Nicola Silvestris, Roberto Salvia, Aldo Scarpa and Claudio Luchini

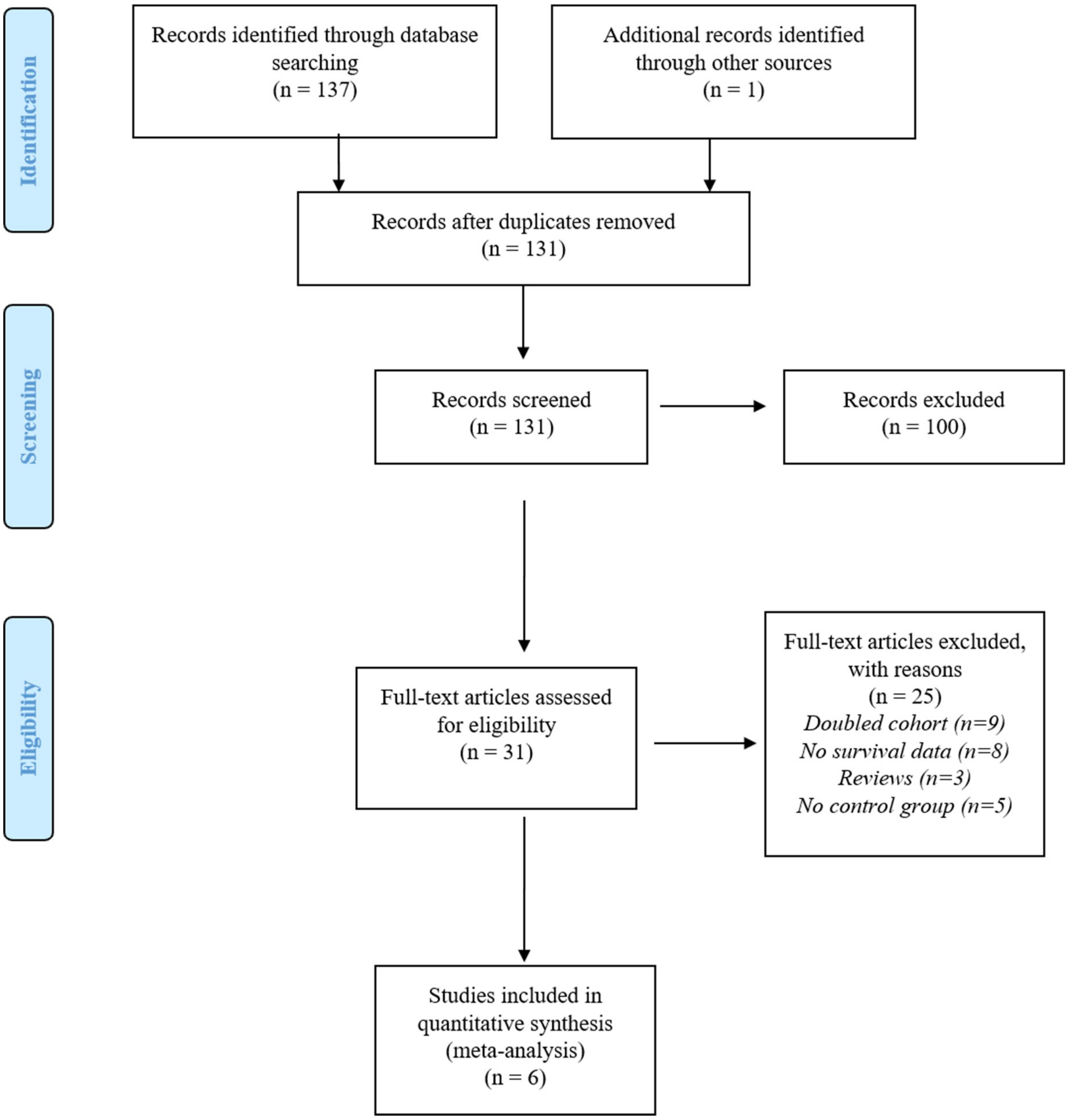

Figure 1. PRISMA checklist for this meta-analysis.

Study name	Statistics for each study				
	Risk ratio	Lower limit	Upper limit	Z-Value	p-Value
Karamitopoulou et al., 2013	3,862	1,978	7,541	3,958	0,000
Lohneis et al., 2018	1,153	1,010	1,316	2,112	0,035
Liu et al., 2017	1,425	1,094	1,856	2,625	0,009
	1,610	1,048	2,474	2,175	0,030

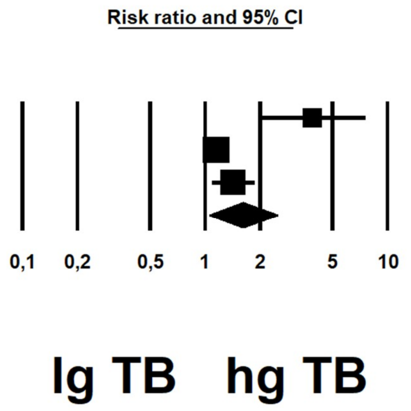

Figure S2. Forrest plot indicating pancreatic ductal adenocarcinoma risk ratio for recurrence in patients with high-grade tumor budding vs. low-grade tumor budding.

Table S1. Characteristics of the studies according to tumor budding, high-grade (Hg-TB) vs. low grade (Lg-TB).

				**Hg-TB**					**Lg-TB**							
**Study Author, Year (Country)**	**Exclusion Criteria**	**Methods of TB Assessment**	**Analysis of EMT-associated variables**	**Number of Participants**	**N. of Females (%)**	**Mean Age ± SD**	**pT**	**LNM**	**Number of Participants**	**N. of Females (%)**	**Mean Age ± SD**	**pT**	**LNM**	**Number of Adjustments**	**NOS**	**Mean Follow-Up Period (Months)**
Chouat, 2018 (Tunisia) [1]	M1	1	cytokeratin, vimentin	28	NA	NA	NA	NA	22	NA	NA	NA	NA	4	8	34
Karamitopoulou, 2013 (Greece) [2]	NS	2	NA	83	45.8%	68 (44-84)	pT1–2: 6.4%; pT3–4: 93.6%	No: 17.1%; Yes: 82.9%	34	44.1%	65 (range: 34-83)	pT1–2: 18.2%; pT3–4: 81.8%	No: 21.1%; Yes: 78.8%	4	8	>36
Liu, 2017 (China) [3]	R2 resection, M1, DPC	1	Expression of cytokeratin	20	35.3%	60.8±11.7	pT1–2: 17.6%; pT3–4: 82.4%	No: 35.3%; Yes: 64.7%	26	37.9%	60.9±11.2	pT1–2: 10.3%; pT3–4: 89.7%	No: 37.9%; Yes: 62.1%	0	6	NS
Lohneis, 2018 (Germany) * [4]	Lack of follow-up information	3	NA	57	67%	<65 y: 58%; ≥65 y: 42%	pT1–2: 14%; pT3–4: 86%	No: 25%; Yes: 75%	116	55%	<65 y: 66%; ≥65 y: 34%	pT1–2: 10%; pT3–4: 90%	No: 24%; Yes: 76%	7	8	>36
O'Connor, 2015 (Canada) [5]	NS	2	NA	32	NA	NA	NA	NA	136	NA	NA	NA	NA	0	6	>36
Zhang, 2016 (China) [6]	No diagnosis of PDAC	4	NA	31	NA	NA	NA	NA	28	NA	NA	NA	NA	2	6	15
**Total Studies (weighted values)**	-	**1, 2: 2 studies; other: 1 study each**	**Vimentin: 1 study**	**251**	**51.9%**	**66.6 y**	**pT1–2: 10.7%; pT3–4: 89.3%**	**No: 21.9%; Yes: 78.1%**	**362**	**50.6%**	**63.2 y**	**pT1-2: 11.9%; pT3-4: 88.1%**	**No: 25.6%; Yes: 74.4%**	**Range: 0–7**	**Median: 7**	**Mean: 31.4**

Abbreviations: EMT: epithelial.to-mesenchymal transition; M1: metastatic disease; NS: not specified; NA: not available/ not assessed; DPC: death for post-operative complications; PDAC: pancreatic ductal adenocarcinoma; pT pathologic stage; LNM: lymph node metastasis.

Methods of tumor budding assessment:

(1) Tumor budding was defined according to the consensus definition as the presence of de-differentiated single cells or small clusters of up to 5 cells in the tumor stroma (at the center or the periphery of tumor). More than 10 high-power field (HPF) were examined. The HPF surface was 0.19 mm^2. Tumor budding were categorized into low-grade (if 1 HPF comprised between 1 and 9 buds) and high-grade (if 1 HPF contained > 10 buds);

(2) Tumor budding was defined according to the consensus definition as the presence of de-differentiated single cells or small clusters of up to 5 cells in the tumor stroma (at the center or the periphery of tumor). The number of buds was counted using the 40× magnification (surface 0.49 mm^2) in 10HPFs (400×) and the scoring was performed based to the average number of buds. Cases with an average of 0–10 buds across 10 HPFs were defined as low-grade budding, while cases with an average of >10 buds across 10 HPFs were defined as high-grade budding;

(3) The quantification approach published by ITBCC for reporting tumor budding in colorectal cancer was used. Therefore, tumor buds were counted in one field of view (20× objective, 22 mm field of view ocular) at the "hotspot" of budding and the number of tumor buds per 0.785 mm^2 was determined using a normalization factor. Budding was grouped according to ITBCC into Bd 1 (0–4 buds), Bd 2 (5–9 buds) and Bd 3 (10 or more buds). Bd 1 and Bd 2 formed the category of low-grade tumor budding, while Bd 1 was considered as high-grade tumor budding. Additionally, in areas of maximal tumor budding, detected at scanning magnification, the number of tumor buds was counted in 10 high-power fields (1 HPF 0.238 mm^2, 40× objective; 22mm field of view ocular). Tumor budding was not classified into peritumoral and intratumoral;

(4) Tumor budding was defined according to the consensus definition as the presence of de-differentiated single cells or small clusters of up to 5 cells in the tumor stroma (at the center or the periphery of tumor). More than 10 high-power field (HPF) were examined. The HPF surface was 0.19 mm^2. Tumor budding were categorized into low-grade (if 1 HPF comprised between 1 and 17 buds) and high-grade (if 1 HPF contained > 17 buds).

Table 2. Methodological quality of cohort studies included in the meta-analysis*.

First Author, Publication Year	Representativeness of the Exposed Cohort	Selection of the Unexposed Cohort	Ascertainment of Exposure †	Outcome of Interest Not Present at Start of Study ††	Control for Important Factor or Additional Factor †††	Assessment of Outcome	Follow-Up Long Enough for Outcomes to Occur ††††	Adequacy of Follow-Up of Cohorts	Total Quality Scores
Chouat, 2018 [1]	*	*	*	*	**	*	-	*	8
Karamitopoulou, 2013 [2]	*	*	*	*	*	*	*	*	8
Liu, 2017 [3]	*	*	*	*	-	*	-	*	6
Lohneis, 2018 [4]	*	*	*	*	*	*	*	*	8
O'Connor, 2015 [5]	*	*	*	-	-	*	*	*	6
Zhang, 2016 [6]	*	*	*	-	*	*	-	*	6

Original studies were analyzed in the quality assessment.

* A study could be awarded a maximum of one star for each item except for the item Control for important factor or additional factor. The definition/explanation of each column of the Newcastle-Ottawa Scale is available at http://www.ohri.ca/programs/clinical_epidemiology/oxford.htm.

† For this index, one star was given if in Method section tumor budding assessment was clearly defined (e.g.: microscopic definition and method of count of buds)

†† Being outcome of interest mortality, we took as outcome of interest for assessment of quality if the risk of recurrence was assessed.

††† A maximum of 2 stars could be awarded for this item. Studies that controlled their survival analyses for at least two confounders received one star, whereas studies that investigated also epithelial-to-mesenchymal transition markers, an additional star.

†††† A cohort study with a mean/median follow-up time ≥3 y (36 months) takes one star.

Table 3. Type and number of adjustments (in addiction of tumor budding) for each study.

First author, Publication Year	Adjustments	Maximum Number of Adjustments
Chouat, 2018 [1]	Age, tumor size, R status, vimentin expression	4
Karamitopoulou, 2013 [2]	R status, pN, L status, chemotherapy	4
Liu, 2017 [3]	-	0
Lohneis, 2018 [4]	Treatment, pT, pN, G, R status, age, sex	7
O'Connor, 2015 [5]	-	0
Zhang, 2016 [6]	AJCC, parasympathetic neurogenesis	2

Abbreviations: R status: status of resection margins, pT: pathologic tumor stage; pN: pathologic status of lymph nodes; G: tumor grading, AJCC: comprehensive stage according to the American Joint Commission on Cancer staging system.

Reference

1. Chouat, E.; Zehani, A.; Chelly, I.; Njima, M.; Maghrebi, H.; Bani, M.A.; Njim, L.; Zakhama, A.; Haouet, S.; Kchir, N. Tumor budding is a prognostic factor linked to epithelial mesenchymal transition in pancreatic ductal adenocarcinoma. Study report and literature review. *Pancreatology* **2018**, *18*, 79–84, doi:10.1016/j.pan.2017.11.010.
2. Karamitopoulou, E.; Zlobec, I.; Born, D.; Kondi-Pafiti, A.; Lykoudis, P.; Mellou, A.; Gennatas, K.; Gloor, B.; Lugli, A. Tumour budding is a strong and independent prognostic factor in pancreatic cancer. *Eur. J. Cancer* **2013**, *49*, 1032–1039, doi:10.1016/j.ejca.2012.10.022.
3. Liu, D.N.; Lv, A.; Tian, Z.H.; Tian, X.Y.; Guan, X.Y.; Dong, B.; Zhao, M.; Hao, C.Y. Superior mesenteric artery margin in pancreaticoduodenectomy for pancreatic adenocarcinoma. *Oncotarget* **2017**, *8*, 7766–7776, doi:10.18632/oncotarget.13950.
4. Lohneis, P.; Sinn, M.; Klein, F.; Bischoff, S.; Striefler, J.K.; Wislocka, L.; Sinn, B.V.; Pelzer, U.; Oettle, H.; Riess, H.; et al. Tumour buds determine prognosis in resected pancreatic ductal adenocarcinoma. *Br. J. Cancer* **2018**, *118*, 1485–1491, doi:10.1038/s41416-018-0093-y.
5. O'Connor, K.; Li-Chang, H.H.; Kalloger, S.E.; Peixoto, R.D.; Webber, D.L.; Owen, D.A.; Driman, D.K.; Kirsch, R.; Serra, S.; Scudamore, C.H.; et al. Tumor budding is an independent adverse prognostic factor in pancreatic ductal adenocarcinoma. *Am. J. Surg. Pathol.* **2015**, *39*, 472–478, doi:10.1097/PAS.0000000000000333.
6. Zhang, L.; Guo, L.; Tao, M.; Fu, W.; Xiu, D. Parasympathetic neurogenesis is strongly associated with tumor budding and correlates with an adverse prognosis in pancreatic ductal adenocarcinoma. *Chin. J. Cancer Res.* **2016**, *28*, 180–186, doi:10.21147/j.issn.1000-9604.2016.02.05.

© 2019 by the authors. Licensee MDPI, Basel, Switzerland. This article is an open access article distributed under the terms and conditions of the Creative Commons Attribution (CC BY) license (http://creativecommons.org/licenses/by/4.0/).
